# Topically Applied Molecular Hydrogen Normalizes Skin Parameters Associated with Oxidative Stress: A Pilot Study

**DOI:** 10.3390/antiox14060729

**Published:** 2025-06-14

**Authors:** Natalia Debkowska, Marek Niczyporuk, Arkadiusz Surazynski, Katarzyna Wolosik

**Affiliations:** 1 Independent Cosmetology Laboratory, The Faculty of Pharmacy, Medical University of Bialystok, Kilinskiego 1, 15-089 Bialystok, Poland; natalia-d-pocztawp@wp.pl; 2 Department of Aesthetic Medicine, The Faculty of Pharmacy, Medical University of Bialystok, Kilinskiego 1, 15-089 Bialystok, Poland; marek.niczyporuk@umb.edu.pl; 3 Department of Medicinal Chemistry, The Faculty of Pharmacy, Medical University of Bialystok, Kilinskiego 1, 15-089 Bialystok, Poland; arkadiusz.surazynski@umb.edu.pl

**Keywords:** molecular hydrogen, oxidative stress, antioxidant

## Abstract

Topical application of molecular hydrogen (H_2_) has recently emerged as a promising strategy to counteract oxidative stress-related skin damage. This pilot clinical study aimed to assess the efficacy of hydrogen-rich water treatments in improving objective skin parameters in healthy adults. The hypothesis was that H_2_, through its selective antioxidant and anti-inflammatory properties, would reduce oxidative stress, modulate inflammatory pathways, and enhance skin barrier integrity, leading to measurable improvements in skin appearance. Fifteen participants received topical treatments with hydrogen-rich water for four weeks. Skin parameters, including porphyrin levels, pigmentation irregularities, pore size, wrinkle severity, and biological skin age, were quantitatively assessed before and one week post-treatment. A statistically significant reduction in pore visibility was observed, particularly in younger participants. Although porphyrin levels showed a trend toward reduction, this change was not statistically significant. Improvements were also noted in pigmentation, wrinkle severity, and estimated biological skin age. The treatment was well tolerated, with no adverse effects reported. Despite promising outcomes, this study was limited by the absence of a control group and a relatively short follow-up period. Further controlled studies with larger sample sizes and molecular biomarker analyses are needed to confirm these effects and elucidate the underlying mechanisms. This study addresses a gap in the literature regarding standardized, clinical evaluation of topical H_2_ application and highlights its potential for utilization in cosmetic and preventive dermatology.

## 1. Introduction

Skin is a primary target of oxidative stress, a process characterized by an imbalance in the production of reactive oxygen species (ROS) and the capacity of antioxidant defenses to neutralize them. Excessive ROS generation, caused by UV radiation, pollution, and stress, contributes to oxidation of skin lipids, proteins, and DNA, leading to damage of skin cells and skin aging signs like wrinkles, pigmentation, enlarged pores, and barrier dysfunction [1,2,3]. These oxidative insults affect both the superficial epidermal layers and deeper dermal structures, disrupting skin homeostasis. Although the skin possesses endogenous antioxidant systems, such as catalase, superoxide dismutase, glutathione peroxidase, and vitamins C and E, their efficiency declines with age and chronic exposure to environmental stress, making exogenous antioxidant strategies relevant [4]. This highlights the ongoing need for effective and selective antioxidant interventions that support skin defense mechanisms and promote repair.

In recent years, molecular hydrogen (H_2_) has emerged as a novel and promising antioxidant molecule in dermatological and systemic medicine. This small, neutral, non-polar diatomic gas selectively scavenges highly reactive radicals, including hydroxyl radicals (•OH) and peroxynitrite (ONOO⁻), without affecting beneficial species such as superoxide anion (O_2_•⁻) and hydrogen peroxide (H_2_O_2_) that are essential for physiological signaling [1,5]. Due to its low molecular weight (2.016 g/mol), H_2_ can rapidly diffuse across cell membranes, penetrating the stratum corneum and reaching subcellular compartments such as mitochondria and nuclei, which mitigates oxidative stress at its source [6,7]. Unlike conventional antioxidants, which may become unstable or pro-oxidative at high concentrations, molecular hydrogen’s only byproduct is water, making it biologically safe and metabolically neutral [6]. Beyond dermatological applications, molecular hydrogen has shown therapeutic potential in mitigating oxidative stress–induced degeneration in other highly metabolic and oxygen-sensitive tissues, such as the retina. Retinal pigment epithelial (RPE) cells, which play a crucial role in photoreceptor maintenance and visual cycle homeostasis, are particularly susceptible to damage from ROS. Accumulating evidence suggests that H_2_ attenuates oxidative retinal injury by decreasing lipid peroxidation, DNA fragmentation, and inflammation, thereby offering protection in experimental models of retinal degeneration and age-related macular degeneration [8]. These findings illustrate the cross-tissue antioxidant efficacy of molecular hydrogen and support its clinical relevance in modulating redox homeostasis, not only in ocular health but also in skin, another ROS-vulnerable organ constantly exposed to environmental stressors. This broader biological context provides a strong rationale for examining topical H_2_ as a selective antioxidant intervention in the skin. The beneficial effects of molecular hydrogen are not limited to skin or ocular structures. A recent systematic review and meta-analysis confirmed that hydrogen-rich water (HRW) significantly reduces inflammatory cytokines (IL-1β, IL-6, and TNF-α), oxidative stress markers (e.g., 8-hydroxydeoxyguanosine), and the activity of pathogenic oral bacteria in patients with periodontal disease [9]. Moreover, it increased glutathione peroxidase activity, suggesting that H_2_ not only scavenges ROS but also supports endogenous antioxidant systems. These findings emphasize hydrogen’s anti-inflammatory and redox-modulating capabilities in epithelial and connective tissues vulnerable to chronic oxidative damage—features that are directly relevant to skin physiology and aging.

Further supporting its systemic relevance, molecular hydrogen has demonstrated potential as an adjunct therapy in oncology. In a randomized, placebo-controlled clinical trial, patients undergoing radiotherapy for liver tumors who consumed HRW reported improved quality of life, reduced fatigue, and lower circulating levels of oxidative metabolites, without compromising the antitumor effects of the treatment [10]. This selective protection against ROS-induced damage, without interfering with necessary cytotoxic mechanisms, positions H_2_ as a unique and biologically compatible antioxidant agent.

Extending beyond peripheral tissues, H_2_ has also been shown to exert beneficial effects on the central nervous system. In a double-blind crossover study, healthy adults consuming HRW experienced reductions in anxiety and mental fatigue and improved autonomic nervous system balance, as evidenced by changes in sympathetic activity at rest [11,12]. These results suggest that H_2_ may improve psychological resilience and quality of life through modulation of neuro-oxidative stress and inflammatory signaling. Given the well-established links between psychological stress, skin barrier disruption, and inflammatory dermatoses, such effects further justify the exploration of molecular hydrogen in skin-focused interventions. Given the bidirectional communication between the brain and the skin, referred to as the brain–skin axis, the psychological benefits of H_2_ may indirectly enhance skin health, particularly under stress-related conditions such as acne, rosacea, or inflammatory dermatoses.

These findings underscore the multifaceted biological effects of H_2_, which extend beyond ROS scavenging to include anti-inflammatory, neuroprotective, and systemic resilience-enhancing properties. This broader biological context strengthens the rationale for examining topical hydrogen applications as selective, safe, and physiologically adaptive antioxidant strategies for maintaining skin homeostasis. Moreover, by potentially mitigating stress-related oxidative and inflammatory processes, topical H_2_ may also influence skin conditions linked to psychological triggers.

Beyond its well-established role in redox regulation, molecular hydrogen also modulates key processes involved in skin integrity and aging. It influences collagen homeostasis, suppresses UV-induced melanogenesis, and downregulates matrix metalloproteinase (MMP) activity, thereby limiting the degradation of dermal extracellular matrix components under chronic oxidative stress [1]. These combined effects highlight the potential of molecular hydrogen as a skin-rejuvenating, anti-inflammatory, and photoprotective agent.

From a dermatological perspective, hydrogen-rich water provides an effective, targeted treatment option that avoids systemic absorption. When used in practices, such as baths and facial cleansing routines, it has been shown to reduce sebum, porphyrins, inflammatory acne lesions, and pigmentation irregularities, improving skin condition and appearance [13,14,15,16].

Moreover, H_2_ modulates redox-sensitive signaling pathways, such as activating nuclear factor erythroid 2–related factor 2 (Nrf2), which upregulates the transcription of endogenous antioxidant and cytoprotective enzymes (e.g., heme oxygenase-1 [HO-1], NAD(P)H quinone oxidoreductase 1 [NQO1], and glutathione S-transferase) and downregulating nuclear factor kappa B (NF-κB), reducing the transcription of pro-inflammatory cytokines (e.g., TNF-α, IL-1β, and IL-6) [6]. This dual action of H_2_, both antioxidant and anti-inflammatory, makes it a compelling candidate for interventions aimed at reducing oxidative stress and inflammation in the skin [6].

Despite its promising biological properties, the topical application of molecular hydrogen remains unexplored, particularly in clinical settings. Most of the available data originates from preclinical or in vitro studies, and there is a lack of standardized, quantitative assessments of its effects on skin parameters in human populations. Furthermore, the impact of topical H_2_ application on biological skin age, a marker of cumulative oxidative damage and regenerative potential, has not yet been investigated.

We hypothesize that the selective antioxidant and anti-inflammatory properties of molecular hydrogen, when applied topically in a standardized manner, will result in measurable improvements in skin parameters (e.g., pigmentation, porphyrin levels, pore size, wrinkle severity, and biological skin age), primarily by reducing oxidative stress, modulating inflammatory pathways, and restoring skin barrier integrity.

This pilot clinical study was designed to address this hypothesis by assessing the effects of topical H_2_ on objectively measured skin parameters in healthy adults across different age groups.

## 2. Materials and Methods

### 2.1. Participants in This Study

A prospective, single-arm, pilot clinical study was conducted at the Medical University of Bialystok (Poland) between September 2024 and May 2025. The objective of this study was to investigate the impact of topically applied molecular hydrogen on biophysical skin parameters. This study was approved by the Human Research Ethics Committee of the Medical University of Bialystok (approval No. APK.002.296.2024) and was conducted in accordance with the principles of the Declaration of Helsinki.

Prior to enrollment, written informed consent was obtained from all participants, including consent for the use of photographic documentation for publication purposes. An assessment of biophysical skin parameters was conducted at three time points, employing non-invasive instrumental methodologies. The subjects were informed that they could withdraw from this study at any time without providing a reason. Participants were excluded from this study if they met any of the following criteria: pregnancy or lactation; viral, fungal, or bacterial skin infections; active herpes simplex lesions; open wounds, fresh scars, or a history of surgical procedures in the treatment area within the past six months; current or recent (within six months) systemic isotretinoin therapy; or known immunosuppression. One participant in this study had a documented history of atopic dermatitis (AD); however, this participant was in clinical remission and exhibited only signs of impaired skin barrier function. As having AD in remission without active lesions did not constitute an exclusion criterion or pose a contraindication to treatment with molecular hydrogen, this participant was included in this study.

The study population consisted of 15 adults divided into three age groups. Group 1 (I—young adults) included seven individuals aged between 21 and 26 years (mean age: 23 years). Group 2 (II—middle-aged adults) comprised four participants aged between 33 and 45 years (mean age: 42 years). Group 3 (III—older adults) consisted of four individuals aged between 55 and 72 years (mean age: 64 years).

### 2.2. Apparatus for Molecular Hydrogen Application

The application of topical molecular hydrogen was carried out using the Hebe Hydrogenium+ device (Hebe, Poland), which has been specifically engineered for non-invasive hydrogen application procedures to enhance cutaneous condition. The Hydrogenium+ system consists of two chalices. Chalice 1 contains two compartments created by a nanosilver-stabilized, water-insoluble membrane. This chalice includes two titanium electrodes coated with a 25-micron layer of platinum, enabling the electrolysis process. One of these electrodes, responsible for producing alkaline water, is connected to an electrolysis generator and intake pipes, which allow real-time monitoring of the alkaline water condition. Chalice 2 collects the used water after treatment. Low-mineral water (with a mineral content of less than 500 mg/L) is added to Chalice 1 before each treatment. The device is activated via the control panel and reaches operational readiness when the display indicates 100%. During electrolysis, the generation of alkaline water occurred on one side of the membrane, while the other side yielded acidic water comprising chloride and sulfate ions. It is important to note that solely the alkaline fraction was employed for cutaneous application. The application of hydrogen water to the skin was conducted through a specialized treatment tip, with the process being executed under strictly controlled pressure conditions. All treatments were carried out by the same trained operators under standardized conditions.

### 2.3. Assessment of Alkaline Hydrogen-Rich Water Parameters

The parameters of the alkaline hydrogen-rich water used in the procedure were evaluated using a multi-functional water quality meter (Vigomed, Poland) capable of measuring pH, oxidation–reduction potential (ORP), molecular hydrogen (H_2_) concentration, and water temperature. The primary purpose of this assessment was to ensure consistency and the antioxidative potential of the hydrogenated water applied to the skin.

The pH of the water was measured with a range of 0.01 to 14.00 pH units, with a resolution of 0.01 and an accuracy of ±0.05 pH. The oxidation–reduction potential (ORP) was measured to assess the antioxidative capacity of the water. ORP values reflect the redox balance of the water—more negative values are associated with higher reducing (antioxidant) power. The ORP measurement range was ±999 mV, with a resolution of 1 mV and an accuracy of ±2 mV.

The hydrogen concentration in the water was evaluated as a key determinant of its biological effectiveness. The measurement range for molecular hydrogen was 0–2400 ppb (equivalent to 0.001–2.400 ppm), with a resolution of 2 ppb or 2 ppm and an accuracy of ±10 ppb or ±10 ppm. Water temperature was monitored during each session to ensure reproducibility and standardization of the treatment. Temperature was recorded in the range of 0.1–60.0 °C (32.0–140 °F), with a resolution of 1 °C/°F and accuracy of ±0.5 °C.

All measurements were conducted prior to each session to verify the quality and stability of the hydrogen-rich water used in the procedures.

### 2.4. Treatment Procedure

The measurement protocol was adapted from the methodology previously described by Chilicka et al. [15]. The standardized treatment procedure involved a consistent sequence of steps designed to ensure procedural reproducibility and participant safety. Each session commenced with preparation of the treatment station in accordance with hygiene and safety regulations, followed by the arrangement of all necessary materials and instruments. Prior to the first session, each participant underwent a detailed interview and completed an informed consent form, acknowledging their understanding of the procedure and granting permission for photographic documentation.

Before the first treatment, participants’ facial skin was thoroughly cleansed, followed by a 10-minute acclimatization period to allow for skin stabilization. Standardized diagnostic photographs of the treatment area were taken before the initial procedure. Each participant received four treatment sessions, scheduled at one-week intervals. Follow-up assessments were conducted immediately after the final session and again seven days later to evaluate the short-term effects of the treatment.

Each treatment session consisted of two sequential stages. First, the facial skin was cleansed using a micellar cleansing gel to remove makeup and surface impurities. Hydrogen application was performed using the H_2_ Peel handpiece, which delivered hydrogen-rich water while simultaneously generating a vacuum that gently lifted the skin fold. One conduit supplied clean water, while a second conduit removed the used hydrogenated water and directed it to a waste container. The suction power was consistently set at approximately 10% during all four sessions to minimize the risk of bruising. This stage lasted 10 min and was performed uniformly across all participants.

The second stage involved the use of the H_2_ Jet handpiece to deliver pressurized hydrogen-rich water to the skin. This device featured a nozzle that emitted hydrogenated water at a pressure of 2 bar, appropriate for facial applications. The handpiece was moved in sweeping motions approximately 2–3 cm from the skin’s surface for a duration of five minutes. After each treatment session, the skin was allowed to return to baseline temperature during a 10-minute recovery period. Following each session, a moisturizer was applied to the treated area.

Participants were instructed to perform home care that consisted solely of cleansing the face with a micellar gel and applying a moisturizer. They were advised to refrain from using any additional cosmetic products, exfoliating treatments, or device-based facial treatments throughout the study period.

### 2.5. Skin Parameter Assessment

Skin assessments were conducted at three distinct time points: prior to the initial treatment session (baseline), immediately following the final session, and at the 7-day follow-up. Objective evaluations were performed using the Polderma Explore 3D PL system (Polderma, Poland), a multidimensional diagnostic platform for non-invasive dermatological and cosmetological assessment. This device integrates three distinct lighting modalities to enable comprehensive visualization of both superficial and subsurface skin structures. Visible red, green, and blue (RGB) light was employed to assess wrinkles, pores, pigmentation, and surface texture. Ultraviolet (UV) light at 365 nm facilitated the detection of hyperpigmentation and porphyrins associated with sebaceous gland activity. Polarized light (PL) enabled visualization of vascular features (erythematous regions) and melanin-related pigmentation (brown spots). The integrated software supported quantitative analyses and facilitated standardized photographic comparisons over time. The assessments encompassed a comprehensive array of skin characteristics, including but not limited to surface texture, wrinkle depth, sebum content, pore size, pigmentation irregularities, hydration, and microvascular patterns.

The Polderma Explore 3D PL underwent rigorous technical evaluation and received approval from the Supreme Technical Organization (Naczelna Organizacja Techniczna—NOT) in Poland. This verification confirmed compliance with professional standards for measurement accuracy, repeatability, and reliability in clinical and cosmetic skin analysis. This independent validation provides a robust foundation for the scientific integrity of the measurements applied in this study.

### 2.6. Skin Analysis and Quantification Parameters

To quantify the data, the system measures each skin parameter and compares it to a comprehensive reference database, which is categorized by age and sex. Results are expressed as percentages, indicating the participant’s relative position within the normative population. For instance, a result of 67% indicates that 67% of individuals in the same demographic group exhibit similar outcomes, while the remaining 33% demonstrate superior results. All assessments were performed in a controlled room environment by the same trained operators, adhering strictly to the manufacturer’s standardized protocols.

### 2.7. Statistical Analysis

To assess differences in skin parameters across three time points (baseline, immediately after treatment, and 7 days post-treatment), a one-way repeated measures ANOVA was conducted for each variable. This method allowed evaluation of within-subject changes over time. For each analysis, the F-ratio, *p*-value, and partial eta squared (ηp^2^) were reported. A *p*-value of <0.05 was considered statistically significant. In cases where *p* < 0.001, results were considered highly statistically significant, indicating a very low probability that observed differences occurred by chance. The effect size was interpreted according to the following thresholds for ηp^2^: 0.01 = small effect, 0.06 = moderate effect, ≥0.14 = large effect. All participants served as their own controls within a repeated measures design. Results are expressed as means ± standard deviation (SD) unless otherwise stated.

### 2.8. Literature Search and Selection Criteria

A comprehensive literature search was conducted to identify relevant studies on molecular hydrogen’s role in oxidative stress modulation and skin health. Searches were performed in the PubMed, Scopus, and Web of Science databases using the following keywords and combinations: molecular hydrogen, hydrogen therapy, oxidative stress, skin, antioxidant, anti-inflammatory, Nrf2, and NF-κB. The search covered publications from January 2000 to March 2024. The inclusion criteria were as follows: original research articles (preclinical or clinical) related to hydrogen’s effects on skin health and oxidative stress pathways. Peer-reviewed publications available in full text and written in English. Studies reporting molecular mechanisms, clinical applications, or outcomes relevant to topical or systemic hydrogen treatments in dermatology or related fields. Exclusion criteria included non-English publications or abstracts without full-text availability. Articles unrelated to skin health, hydrogen therapy, or oxidative stress. Review articles and meta-analyses were considered for background information but not included in data synthesis. Additionally, reference lists of key papers were screened to identify further relevant studies.

## 3. Results and Discussion

### 3.1. Alkaline Hydrogen-Rich Water Parameters

A multi-parameter water quality meter was used to evaluate the physicochemical properties of both low-mineral water (prior to ionization) and alkaline hydrogen-rich water (after ionization). The meter measured pH, oxidation–reduction potential (ORP), temperature, and the concentration of dissolved molecular hydrogen (H_2_), expressed in both ppb and ppm. All measurements were conducted prior to each session in order to verify the quality and stability of the hydrogen-rich water used in the procedures.

The initial low-mineral water exhibited a neutral pH of 6.71, a positive ORP of +66 mV, and no detectable hydrogen concentration. Following activation in the Hydrogenium+ device, the water exhibited alkaline properties with a pH of 10.38, a strongly negative ORP of −212 mV, and a substantial increase in dissolved hydrogen concentration (2071 ppb/2.091 ppm). The temperature exhibited an increase from 21 °C to a range of 27.2–29.2 °C, indicative of the operational dynamics of the electrolysis system.

Furthermore, separate measurements were performed for the acidic fraction of the ionized water. Acidic water exhibited a pH range of 5.80–6.57 and a highly oxidative ORP of +551 to +555 mV, with no measurable hydrogen content. A comprehensive comparison of these parameters is presented in Table 1 and Table 2. The marked shift in ORP from oxidative to strongly reductive values, along with the high concentration of molecular hydrogen and the significant pH elevation, confirms the antioxidative potential of the treated water and its suitability for topical molecular hydrogen therapy.

The physicochemical parameters of the hydrogen-rich water used in the procedure are consistent with values reported in the literature. Electrolyzed-reduced water (ERW), also known as alkaline hydrogen-rich water, is characterized by an elevated pH (typically ranging from 8.5 to 10.5), a negative oxidation–reduction potential (ORP) of up to −800 mV, and a measurable concentration of dissolved molecular hydrogen ranging from 0.2 to 1.6 ppm, depending on the electrolysis settings and water source [17,18,19]. These properties are considered essential for its antioxidant and biological activity potential. In our study, the hydrogen-rich water generated by the Hebe Hydrogenium+ device demonstrated parameters fully consistent with those reported in the literature, confirming both the quality and reliability of the water used in the procedures. The water exhibited pH values exceeding neutral levels, oxidation–reduction potential (ORP) values below −200 mV, and molecular hydrogen concentrations above 2.0 ppm. These values indicate effective ionization and align closely with data from previous studies investigating the biological activity of electrolyzed-reduced water (ERW), ensuring that the water employed during treatment procedures indeed contained molecular hydrogen.

### 3.2. Disrupted Epidermal Barrier Function and Oxidative Skin Damage

The epidermal barrier is a complex, multifunctional structure primarily located within the *stratum corneum*. It functions as a physical, chemical, immunological, and microbiological shield, thereby protecting the skin from transepidermal water loss (TEWL), microbial invasion, allergens, and environmental stressors such as UV radiation and pollution. A healthy barrier is composed of tightly packed corneocytes embedded in a lipid matrix of ceramides, cholesterol, and free fatty acids. This structure is responsible for maintaining hydration, regulating skin pH, and ensuring controlled permeability [20]. A compromised skin barrier is characterized by a range of symptoms. Irritation is a common feature, as damage to the barrier increases skin sensitivity to external stimuli, resulting in desquamation, burning, stinging, or visible redness. Despite popular belief, desquamation is not primarily caused by dehydration but rather by a tightly controlled epidermal proteolytic cascade. This cascade involves serine proteases, such as the kallikrein-related peptidases (KLKs) KLK5 and KLK7. The activity of these proteases is modulated by the stratum corneum’s pH gradient and endogenous protease inhibitors such as LEKTI. Disruption to this balance can lead to abnormal desquamation [21]. Itching is frequently reported and is caused by the activation of exposed nerve endings through microfissures in the skin surface. It has been hypothesized that a sensation of tightness may arise from cellular dehydration, where keratinocytes undergo contraction and condensation, thereby reducing skin flexibility. Moreover, excessive TEWL has been demonstrated to not only contribute to cutaneous dryness but also to reduce skin elasticity, which can lead to the formation of fine lines and wrinkles [22].

**Figure 1:**
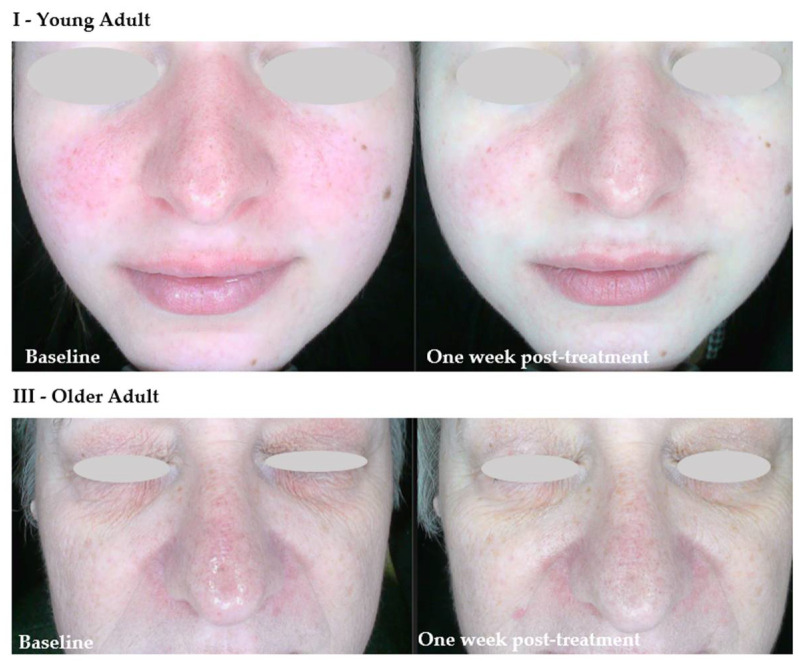
Representative examples of participants from age groups I (young adults) and III (older adults) presenting with features of impaired skin barrier function.

In the present study, following the initial interview and baseline skin assessment prior to the first hydrogen treatment, the predominant finding was the presence of clinical signs indicative of an impaired epidermal barrier. Central facial erythema—most commonly affecting the nose and cheeks—was a predominant feature (Figure 1). Furthermore, some of the participants reported the presence of subjective symptoms, including skin dryness and tightness. In a number of subjects, the skin exhibited signs of desquamation. In some cases, pronounced cutaneous hypersensitivity, particularly in the periorbital region, was observed, thereby further substantiating the impaired barrier function.

The images on the left show the skin condition prior to the initial hydrogen treatment session, while the images on the right illustrate the same subjects seven days after the final session. In Group I, pronounced erythema is visible across the central facial region (cheeks and nose) at baseline, accompanied by mild desquamation around the nasal area and chin. These are indicative of barrier impairment and associated inflammatory response. In the seven days following the conclusion of the treatment regimen, a marked reduction in erythema was observed, accompanied by a discernible enhancement in the texture of the skin. These observations suggest that rehydration has occurred, in addition to a partial restoration of the skin’s barrier integrity. In the older adult participant (III), the baseline image shows dryness, a rough surface texture, and marked desquamation on the nose and periorbital areas, consistent with chronic barrier disruption. The presence of pronounced perinasal scaling and dull skin tone is also observed. Following the administration of hydrogen therapy, the skin exhibits enhanced uniformity and hydrated characteristics, accompanied by substantial softening of desquamative patches and an augmented tone. These observations signify a reparative effect of molecular hydrogen on both acute and age-related barrier dysfunction.

### 3.3. Inflammation and Its Modulation by Molecular Hydrogen

Following the disruption of the epidermal barrier, a secondary but closely interconnected pathological process is the initiation of cutaneous inflammation. The loss of barrier integrity facilitates the transcutaneous penetration of environmental irritants, allergens, and microbial products. These subsequently activate pattern recognition receptors (PRRs) on resident immune and epithelial cells. This immunological alert state leads to the upregulation of nuclear factor kappa B (NF-κB) signaling and downstream transcription of pro-inflammatory cytokines, including interleukin-1β (IL-1β), IL-6, and tumor necrosis factor-alpha (TNF-α). Concurrently, increased vascular permeability, leukocyte infiltration, and the production of ROS perpetuate the inflammatory reaction and amplify tissue injury [6,23].

Molecular hydrogen (H_2_) has been shown to possess anti-inflammatory properties, primarily through its capacity to neutralize ROS, which are pivotal to the initiation and propagation of inflammatory processes in the skin [6]. It has been demonstrated by preceding studies that hydrogen-rich water has the capacity to reduce the expression of pro-inflammatory cytokines and to improve clinical symptoms in dermatological conditions, including atopic dermatitis and psoriasis [16,24,25,26]. A pilot clinical study by Hu et al. [26] explored hydrogen–water bathing (HWB) in patients with atopic dermatitis (AD). Significant reductions in both TEWL and visual analog scale (VAS) scores were observed in areas of the trunk and limbs immersed during bathing over eight weeks. However, areas with minimal water exposure, such as the face and neck, showed less improvement, highlighting the importance of direct hydrogen contact. Alternative delivery methods, such as drinking hydrogen-rich water or applying hydrogen face packs, have been suggested as a way of overcoming limitations in localized application. These strategies could complement HWB, particularly in areas that are less accessible for immersion. Furthermore, hydrogen–water bathing therapy has demonstrated rapid and clinically meaningful improvements in lesion severity and quality of life in patients with chronic inflammatory dermatoses such as psoriasis and parapsoriasis *en plaques* [25]. These results suggest that HWB has the potential to serve as a non-invasive, well-tolerated adjunctive therapy for patients who have had limited success with conventional treatment.

**Figure 2:**
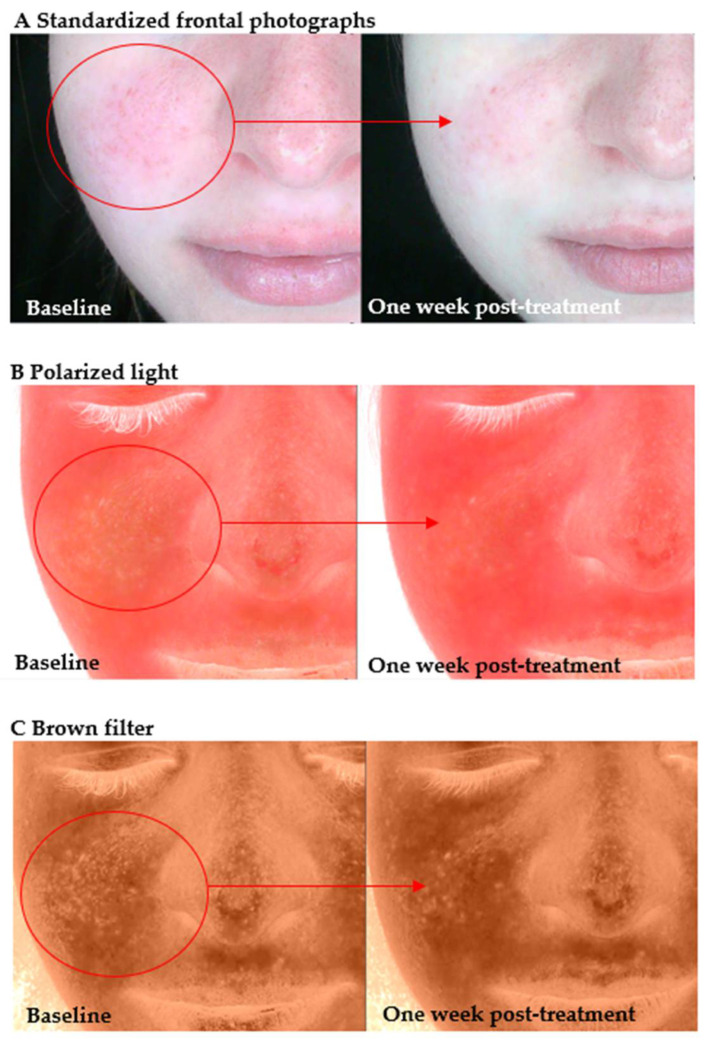
A clinical and imaging evaluation of a participant with atopic dermatitis was conducted before and after a series of molecular hydrogen treatments.

In the present study, following a series of four weekly sessions using molecular hydrogen treatment, a significant reduction in inflammatory and post-inflammatory changes was observed in a participant diagnosed with atopic dermatitis (AD). As demonstrated in Figure 2, visible erythema and inflammatory infiltration were most prominent on the right cheek prior to treatment (A). Following the therapeutic intervention (A), a marked reduction in redness was observed, accompanied by an enhancement in skin tone uniformity. Panel B displays polarized light images that emphasize superficial vascular changes and inflammatory response. Post-treatment evaluation revealed a decrease in overall erythema intensity, suggesting downregulation of inflammation and vascular reactivity. Panel C presents images obtained under the application of a brown filter enhancement, which has been designed to visualize post-inflammatory hyperpigmentation. A reduction in pigment intensity is evident, particularly over the previously inflamed areas, which supports the hypothesis that H_2_ may facilitate not only anti-inflammatory effects but also recovery from pigmentation associated with chronic inflammation. The present findings support the antioxidative and anti-inflammatory properties of molecular hydrogen. In addition, they provide further evidence for its role in restoring epidermal homeostasis in patients presenting with impaired barrier function and AD-like features.

### 3.4. Enlarged Pores and Porphyrins and Molecular Hydrogen Therapy

The presence of enlarged skin pores and porphyrins has been observed in inflammatory skin conditions, such as acne. Porphyrins, which are metabolic by-products of *Cutibacterium acnes*, have been shown to generate ROS upon light exposure, resulting in further skin damage and deterioration of skin condition [27]. Molecular hydrogen has been demonstrated to influence the skin’s microenvironment by limiting the activity of *C. acnes* and the production of porphyrins. A clinical study undertaken over a period of four weeks by Chilicka et al. [15] demonstrated that hydrogen purification treatment, based on the topical application of molecular hydrogen, significantly improved skin condition in women suffering from mild to moderate acne. The observed effects included a reduction in inflammatory lesions, decreased sebum secretion, improved hydration, and overall enhancement of skin appearance and texture. These findings support the potential of molecular hydrogen as a safe adjunctive agent in dermatological practice, particularly due to its anti-inflammatory, antioxidant, and sebum-regulating properties. The proposed mechanism by the authors involves the scavenging of ROS generated by microbial porphyrins, with a particular emphasis on those produced by *Cutibacterium acnes*. Furthermore, the hydrogen-enriched alkaline water utilized in the procedure did not disrupt the physiological skin pH, thereby confirming its tolerability, even among individuals with sensitive or reactive skin [15].

**Figure 3:**
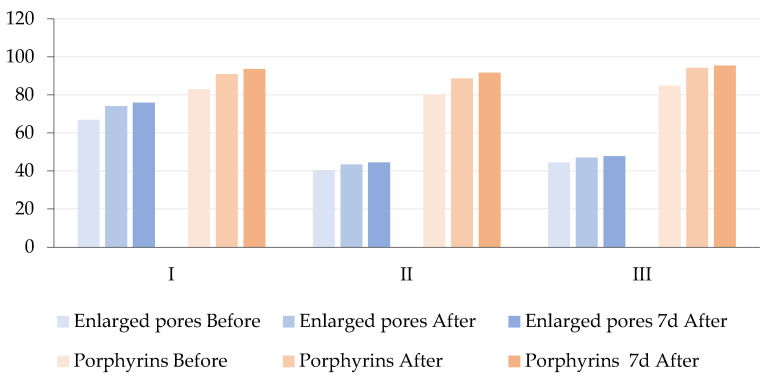
Comparison of mean values for enlarged pores and porphyrins in three age groups (I—young adults, II—middle-aged adults, and III—older adults) across three time points: before treatment (baseline), immediately after hydrogen treatment, and 7 days post-treatment. The chart illustrates the observed decrease in porphyrin fluorescence and the slight modulation of pore visibility, particularly in younger participants.

**Figure 4:**
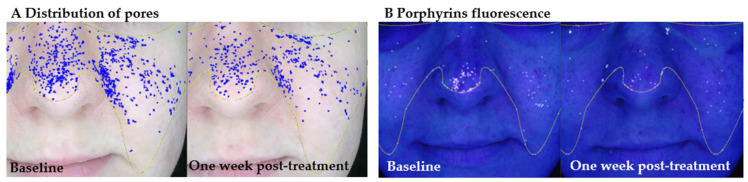
Representative example of a participant from age group I (young adults) presenting with features of follicular congestion and porphyrin accumulation.

In contrast to conventional treatments [28], such as acid-based peels [29,30] or antibiotics, hydrogen purification does not cause irritation or barrier disruption, making it suitable for individuals with impaired skin tolerance. Although not a substitute for dermatological therapies [31], this method has the potential to complement systemic and topical regimens in the management of acne, especially in cases where patients are unable to tolerate pharmacological treatments or where there are present signs of microbial resistance [15]. These results align with our findings of a visible improvement in pore size and porphyrin fluorescence intensity (Table 3 and Figure 3 and Figure 4). The combined evidence supports the role of molecular hydrogen in modulating the skin’s microenvironment, reducing oxidative stress, and improving both the functional and visual parameters of skin affected by inflammation or dysbiosis.

The baseline panels show the distribution of visibly enlarged pores and porphyrin fluorescence, while the right-side panels show the same facial areas seven days after the final session. At baseline, a high density of enlarged pores is visible across the nasal and malar regions, accompanied by porphyrin fluorescence, particularly around the nose and central face. These features are indicative of active sebaceous gland function, microbial colonization, and potential oxidative imbalance within the follicular environment. Seven days following molecular hydrogen therapy, a clear reduction in both the number and density of enlarged pores is observed, along with a decrease in porphyrin fluorescence intensity. These findings suggest that hydrogen therapy may support seboregulation, reduce oxidative stress, and improve follicular clarity in young adult skin.

For pore visibility, the repeated measures ANOVA indicated a statistically significant effect of time, with differences observed both immediately after treatment and one week post-treatment. In the young adult group (M-I), a significant reduction in pore size was observed, with mean values increasing from 66.86 ± 8.45 at baseline to 74.14 ± 10.95 immediately after treatment and 75.86 ± 11.48 one week post-treatment. In the middle-aged group (M-II), a smaller improvement was noted, with mean values rising from 40.50 ± 24.56 at baseline to 43.50 ± 26.66 after treatment and 44.50 ± 27.05 one week later. In the older adult group (M-III), pore size improvements were also observed, with means increasing from 44.50 ± 16.34 at baseline to 47.00 ± 17.42 after treatment and 47.75 ± 18.21 one week post-treatment. The ANOVA revealed a significant effect (F = 5.01, *p* = 0.026, ηp^2^ = 0.455), suggesting that molecular hydrogen treatment effectively reduces pore visibility across all age groups, with the most notable effects observed in younger participants.

Importantly, the pore size and porphyrin fluorescence values presented here are expressed as percentages relative to a normative population, as measured using the Polderma Explore 3D PL system (see Section 2.6 for details). This approach compares each skin parameter with a reference database categorized by age and sex, providing an age-adjusted evaluation of skin condition and treatment progression.

Regarding porphyrins, the baseline level was higher in younger participants and declined with age, consistent with expected physiological trends. In group M-I, porphyrin levels increased from 83.00 ± 5.45 at baseline to 91.00 ± 6.19 after treatment and 93.71 ± 6.50 one week post-treatment. In group M-II, levels increased from 80.25 ± 3.30 at baseline to 88.75 ± 3.59 after treatment and 91.75 ± 3.30 one week later. In group M-III, levels increased from 85.00 ± 0.82 at baseline to 94.25 ± 2.50 after treatment and to 95.50 ± 1.29 one week post-treatment. Although a reduction in porphyrin level was observed after treatment, the repeated measures ANOVA did not reveal statistically significant differences between time points. These findings suggest a trend toward reduced porphyrin levels in all age groups, particularly in younger participants, but without statistical confirmation. The results showed that pore size and porphyrin levels decreased significantly following treatment, with sustained improvements observed one week afterwards. However, the statistical significance of these effects was not maintained at this later time point. The observed decrease in porphyrin levels, a recognized marker of microbial activity, particularly associated with *Cutibacterium acnes*, suggests that molecular hydrogen may contribute to reducing sebum production and improving skin condition overall. These findings are consistent with existing evidence regarding the role of molecular hydrogen in modulating sebum secretion and improving acne-related skin conditions.

Chilicka et al. [15] investigated the effects of four weekly hydrogen purification sessions employing the same device in women with *acne vulgaris* and a control group of healthy participants. They found that sebum levels decreased significantly only in the acne group, while skin hydration increased significantly in both the acne and control groups. This suggests that hydrogen treatments may have both sebum-reducing and moisturizing effects, both of which are important for maintaining skin barrier integrity and alleviating acne symptoms. Furthermore, these results highlight the importance of the alkaline water used in the treatment, as its high pH effectively removes excess sebum from the skin’s surface without causing irritation.

Furthermore, Tanaka and Miwa [16] conducted a study investigating the effects of repetitive bathing and poultice treatments with hydrogen-rich water over periods ranging from 11 to 98 days. They found that hydrogen-rich water had a dual modulatory effect on sebum secretion: it reduced sebum levels in individuals with oily skin and increased them in those with dry skin. The authors reported a negative correlation (r = −0.345) between baseline oiliness levels and the changes observed post-treatment. This indicates that molecular hydrogen has a homeostatic regulatory function in modulating sebum secretion. This modulatory effect is consistent with the reduction in porphyrin levels observed in the present study and supports the hypothesis that hydrogen treatments contribute to the restoration of sebum homeostasis and improvement of the balance of the skin microbiome.

Our study, along with the work of Chilicka et al. and Tanaka and Miwa [15,16], suggests that the ability of molecular hydrogen to modulate sebum secretion may significantly contribute to its beneficial effects on skin health, particularly in conditions such as *acne vulgaris*, where sebum dysregulation plays a central role.

### 3.5. Skin Discolorations: Brown, Red Spots, and UV-Induced Hyperpigmentation

Hyperpigmentation, a common dermatological concern, can be categorized into multiple types based on its etiology and depth. Post-inflammatory erythema (PIE) is defined as red or pink discoloration that arises after inflammation, especially in lighter phototypes, and results from superficial vascular dilation [32]. Post-inflammatory hyperpigmentation (PIH) manifests as brown pigmentation due to elevated melanin production or deposition following cutaneous injury or inflammation [33]. UV-induced pigmentation arises primarily from cumulative exposure to ultraviolet radiation and is associated with melanocyte activity, leading to persistent discoloration [2].

Research findings indicate that oxidative stress and chronic inflammation are pivotal factors in the pathophysiology of hyperpigmentation. According to the research of Xing et al. [34], the generation of ROS by UV exposure has been demonstrated to promote melanogenesis via the stimulation of tyrosinase and pro-inflammatory cytokines. Guo and Zhang’s study [35] demonstrated that hydrogen-rich treatments exhibit antioxidant and anti-inflammatory effects that can mitigate oxidative skin damage, reduce melanin synthesis, and promote skin tone unification through molecular pathways involving Nrf2 activation and suppression of NF-κB-mediated cytokine expression.

**Figure 5:**
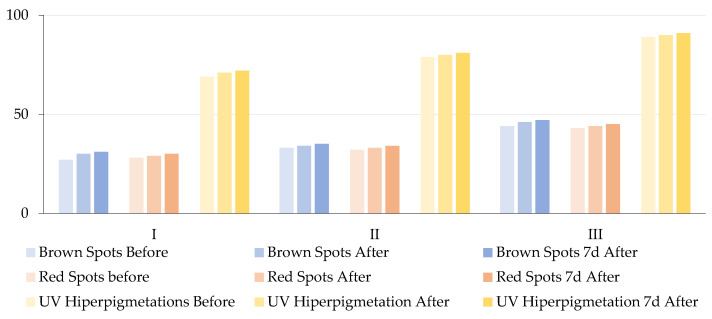
Changes in the mean percentile values of brown spots, red spots, and UV-induced hyperpigmentation in three age groups (I—young adults, II—middle-aged adults, and III—older adults) at three time points: baseline (before treatment), immediately after molecular hydrogen therapy, and 7 days post-treatment.

In the present study, the impact of topical molecular hydrogen application was assessed on three types of hyperpigmented lesions: brown spots, red spots, and UV-related hyperpigmentation, across three age groups. Table 4 and Figure 5 present the results of the repeated measures ANOVA, which was employed to compare baseline values with post-treatment and 1-week follow-up scores. All results are expressed as percentiles, reflecting the patient’s skin condition relative to a normative population matched for age and sex, with higher percentiles indicating better skin condition (i.e., fewer or less intense lesions than in the general population).

Analysis of brown spots revealed a significant increase in percentile values across all three groups. In Group M-I, the percentile improved from 27% at baseline to 30% post-treatment and 31% one week later. Group M-II increased from 33% to 35%, and Group M-III increased from 44% to 47%. These changes were statistically significant (F = 172.64, *p* < 0.001) and had a large effect size (ηp^2^ = 0.8648), which suggests that molecular hydrogen contributed to the reduction in melanin-related pigmentation. Similarly, a significant reduction in red spots was observed, as evidenced by increased percentile values: from 28% to 30% in Group M-I, from 32% to 34% in Group M-II, and from 43% to 45% in Group M-III. These results were also statistically significant (F = 133.49, *p* < 0.001, ηp^2^ = 0.8318), which indicates that molecular hydrogen may exert anti-inflammatory and/or vasoprotective effects. This is consistent with previous findings regarding its ability to suppress pro-inflammatory cytokines and improve microcirculation. Improvements were observed in UV-induced hyperpigmentation: Group M-I demonstrated an increase from 69% to 72%, Group M-II exhibited an increase from 79% to 81%, and Group M-III showed an increase from 89% to 91%. The observed differences were found to be statistically significant (F = 158.57, *p* < 0.001), exhibiting a substantial effect size (ηp^2^ = 0.8545). These findings are consistent with evidence suggesting that molecular hydrogen reduces ROS generated during UV exposure, which are central to melanogenesis and post-inflammatory hyperpigmentation. The results of this study demonstrate that the application of topical molecular hydrogen leads to a statistically significant and clinically relevant improvement in pigmentary skin lesions. This improvement is visible immediately after treatment and is sustained for at least one week post-intervention. The substantial effect sizes across all parameters (ηp^2^ > 0.8) underscore the robustness of the observed changes.

The findings of this study align with emerging evidence on molecular hydrogen’s role in modulating oxidative stress and inflammation in human skin. Hydrogen’s beneficial effects on skin are mediated not only by its direct antioxidant action but also through the modulation of key redox-sensitive signaling pathways. Notably, molecular hydrogen activates the nuclear factor erythroid 2-related factor 2 (Nrf2) pathway, a master regulator of the cellular antioxidant response. Upon activation, Nrf2 translocates to the nucleus and promotes the expression of cytoprotective enzymes, including heme oxygenase-1 (HO-1), NAD(P)H quinone dehydrogenase 1 (NQO1), and glutathione S-transferase (GST), which collectively mitigate oxidative damage and promote tissue homeostasis [1,5].

Furthermore, molecular hydrogen has been shown to inhibit the nuclear factor kappa-light-chain-enhancer of activated B cells (NF-κB) pathway, a key driver of inflammatory responses. By suppressing NF-κB activation, hydrogen reduces the expression of pro-inflammatory cytokines such as tumor necrosis factor alpha (TNF-α), interleukin-1 beta (IL-1β), and interleukin-6 (IL-6), which are implicated in chronic skin inflammation and aging [3].

**Figure 6:**
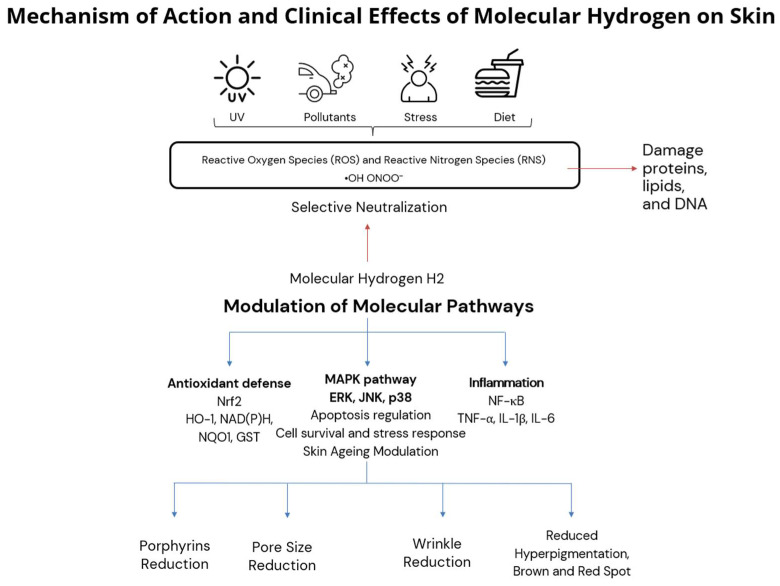
Proposed mechanism of action of molecular hydrogen in skin.

Emerging evidence also suggests that hydrogen modulates mitogen-activated protein kinase (MAPK) signaling, which includes extracellular signal-regulated kinase (ERK), c-Jun N-terminal kinase (JNK), and p38 MAPKs. These kinases regulate cellular responses to oxidative stress, apoptosis, and aging. Preliminary studies indicate that hydrogen may attenuate stress-induced activation of JNK and p38 pathways while supporting ERK-mediated survival signaling, though further research is needed to confirm these effects in human skin models [4]. The proposed mechanism of action of molecular hydrogen in skin is summarized in Figure 6, integrating selective antioxidant and anti-inflammatory pathways with observed clinical effects such as pore size reduction, porphyrin reduction, and improved pigmentation and wrinkle severity.

By modulating these pathways, molecular hydrogen not only reduces oxidative damage but also supports regenerative processes, improves barrier function, and mitigates inflammation in the skin. These mechanistic insights reinforce our clinical findings and suggest that hydrogen-based therapies may offer a multifaceted approach to combating skin aging and oxidative stress, as summarized in Table 5.

In addition to its established antioxidant activity, molecular hydrogen supports tissue repair and remodeling, potentially through mitochondrial protection and modulation of melanogenesis pathways [6]. However, further research is warranted to elucidate these mechanisms and to assess the long-term efficacy of hydrogen-based therapies in clinical settings, particularly for pigmentation disorders. Future studies should include biomarker analyses to correlate clinical outcomes with molecular changes.

### 3.6. Overall Skin Condition, Skin Structure, and Wrinkles

**Figure 7:**
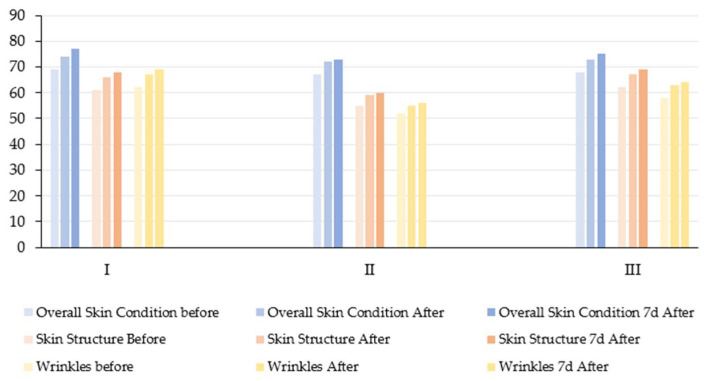
Changes in overall skin condition, skin structure, and wrinkle severity at three time points: baseline (prior to treatment), immediately after the final molecular hydrogen therapy session, and 7 days post-treatment, across three age groups (I—young adults, II—middle-aged adults, and III—older adults).

**Figure 8:**
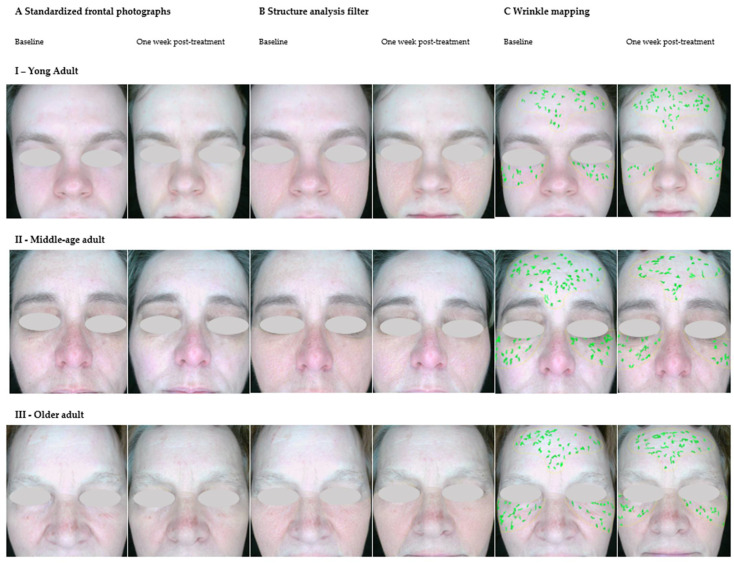
Representative examples of participants from three age groups are provided, including I for young adults, II for middle-aged adults, and III for older adults. Images were captured before the first hydrogen application session (baseline) and seven days after the final session (one week post-treatment). Panels (**A**) show conditions under standard visible light (overall skin condition). Panels (**B**) show a structure analysis filter, where lighter areas indicate greater irregularity and darker areas reflect a smoother, more homogeneous texture. Panels (**C**) show wrinkle mapping, where deeper lines are visualized using increasingly intense green coloring. Improvements were observed in all three age groups, particularly with regard to skin texture regularity and wrinkle visibility. The structural maps show a reduction in light-colored zones, indicating smoother skin post-treatment. Wrinkle density and depth also appear to have decreased, especially in the periorbital and forehead regions.

In the current pilot study, no statistically significant reduction in wrinkle severity was observed in any of the three age groups after undergoing a series of topical molecular hydrogen treatments at room temperature (25 °C) (Table 6, Figure 7 and Figure 8). Although there were visible trends towards improvement (especially in group III, with an increase in mean score from 58.2 to 64.0), the repeated measures ANOVA did not reach statistical significance (*p* > 0.05). However, the effect sizes (e.g., ηp^2^ = 0.2882 for the wrinkle parameter) suggest a moderate practical improvement, which may become more apparent in larger or longer-term studies.

These findings can be compared with those of Tanaka and Miwa [16], who demonstrated a statistically significant reduction in wrinkle depth and severity in five subjects after 11–98 days of daily hydrogen-rich warm water baths (41 °C, 338–682 μg/L H_2_) combined with repeated poultice applications. The authors used a hierarchical wrinkle index scale ranging from − to +++++ and reported a decrease from 3.14 ± 0.52 to 1.52 ± 0.74 (*p* < 0.001), affecting multiple facial areas (eye corners, cheeks, and nasolabial folds). The divergence in results may be attributed to differences in temperature—the warm water (41 °C) likely enhanced transdermal diffusion and vasodilation in this study, intervention lasted up to 98 consecutive days, whereas our protocol included only four weekly sessions. Repeated compress application combined with whole-face immersion probably ensured deeper skin saturation with hydrogen. Nevertheless, both studies support the idea that topical molecular hydrogen treatments can have a positive effect on wrinkle parameters and suggest that longer exposure and thermal enhancement may be necessary to achieve significant remodeling effects in older skin.

The observed improvement in skin structure and reduction in wrinkles following topical molecular hydrogen treatment is biologically plausible and can be attributed to its well-documented antioxidant activity [17,35]. Chronic oxidative stress leads to collagen fragmentation, elastin degradation, and the upregulation of matrix metalloproteinases (MMPs), particularly MMP-1 and MMP-13, which cleave native type I and III collagen fibers in the dermis [36,37]. By reducing oxidative stress, molecular hydrogen may help to preserve collagen and elastin fibers, suppress MMP expression, particularly in UV-exposed or senescent fibroblasts, and stabilize fibroblast metabolic activity. These effects support enhanced biosynthetic functions and the maintenance of the structural integrity of the dermal–epidermal junction (DEJ), which deteriorates progressively with age [35].

### 3.7. Skin Age vs. Chronological Age Before and After Hydrogen Therapy

**Figure 9:**
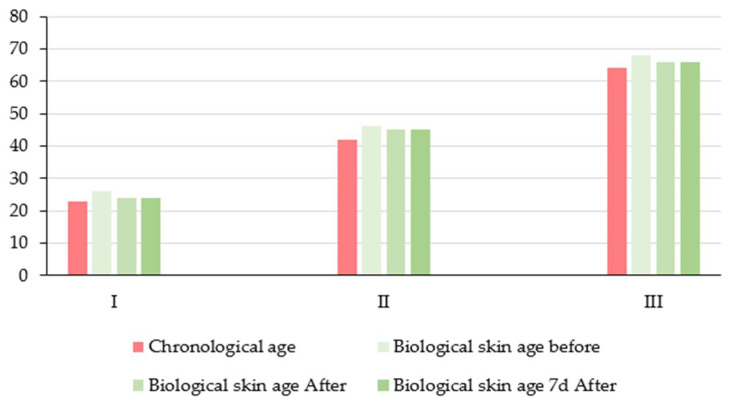
Comparison of mean chronological age and estimated biological skin age in three age groups (I—young adults, II—middle-aged adults, and III—older adults) at three time points: before treatment (baseline), immediately after molecular hydrogen (H_2_) therapy, and 7 days post-treatment.

In order to evaluate the potential anti-aging effects of topically applied molecular hydrogen (H_2_) therapy, a comparison was made between the mean chronological age of participants in each age group and their mean biological skin age, as assessed via digital skin analysis (Figure 9). The estimated skin age is determined by the overall dermatological condition, which is assessed based on various parameters including, but not limited to, texture, elasticity, hydration, pigmentation, and wrinkle depth. In Group I (mean chronological age: 23 years), the mean biological skin age prior to treatment was 26 years, indicating a slight acceleration of skin aging relative to chronological age. Following H_2_ therapy, a decrease in skin age to 24 years was observed, and this value remained stable at the 7-day follow-up, indicating a quantifiable rejuvenation effect and enhanced skin function in young adult skin. In Group II (mean chronological age: 42 years), the skin age decreased from 46 to 45 years following hydrogen therapy, with no further change after one week. Although the improvement was subtle, it suggests a positive response of middle-aged skin to hydrogen’s antioxidant and restorative properties. In Group III (chronological age: 64 years), the skin age was reduced from 68 to 66 years, with the effect sustained after a period of seven days. This result suggests that molecular hydrogen may help to attenuate age-related skin deterioration and support structural skin integrity in older individuals.

Across all age groups, topically applied molecular hydrogen therapy resulted in a reduction in average biological skin age, indicating improvements in dermatological health and visual appearance. The most marked relative benefit was observed in the youngest group, presumably due to higher regenerative potential. These findings lend support to the hypothesis that molecular hydrogen exerts a cytoprotective and rejuvenating effect through the modulation of oxidative stress, free radical neutralization, and restoration of skin barrier function.

### 3.8. Limitations and Context-Dependent Effects of Molecular Hydrogen

Although many studies confirm the antioxidative and anti-inflammatory effects of molecular hydrogen, findings remain context-dependent. Variability in study design, delivery method (e.g., bathing, topical, inhalation), and treatment duration often yields inconsistent results. Kawamura et al. emphasize the need for standardized, long-term clinical trials—particularly in dermatology [38]. This variability is also evident in other fields. In patients with COVID-19 and chronic pulmonary conditions, hydrogen inhalation has been shown to improve respiratory function and reduce inflammatory markers, suggesting benefits for systemic inflammation management. In elderly subjects, long-term ingestion of hydrogen-rich water supported telomere maintenance and lipid metabolism, indicating a possible role in mitigating aging-related decline [39]. In the context of exercise physiology, a meta-analysis of 27 trials (*n* = 597) showed that hydrogen supplementation slightly improved lower-limb power and reduced perceived exertion but had no significant impact on aerobic or anaerobic capacity [40]. A complementary review on oxidative stress markers in healthy adults found no effect on d-ROMs but a modest increase in antioxidant potential [39]. These results suggest hydrogen may support resilience rather than act as a universal performance enhancer. Our own results and other dermatological studies show benefits in hydration and skin tone, yet findings across trials remain inconsistent. For example, Huang et al. (2022) reviewed studies where hydrogen-rich baths improved skin texture and reduced wrinkles but also noted other trials with inconclusive outcomes [41]. Such discrepancies may result from differences in concentration, application time, skin condition, or assessment methods. Together, these findings underscore the need for individualized, context-aware hydrogen therapy protocols tailored to physiological status, age, and treatment goals.

## 4. Conclusions

This prospective, single-arm pilot study demonstrates that topical application of molecular hydrogen (H_2_), delivered via hydrogen-rich water, leads to measurable improvements in key dermatological parameters across different age groups. Statistically significant enhancements were observed in pigmentation-related features, including brown spots, red spots, and UV-induced hyperpigmentation. The findings indicate a strong and reproducible clinical benefit of H_2_ therapy in improving skin tone uniformity and mitigating photo-induced skin changes.

Furthermore, a decline in porphyrin fluorescence and an increase in pore visibility, particularly among younger subjects, indicate potential effects on seboregulation and, conceivably, antimicrobial activity. Although wrinkle depth and structural skin features did not reach statistical significance, moderate effect sizes and visible improvements suggest a trend toward long-term anti-aging benefits, especially with extended or repeated treatments. A decrease in estimated biological skin age was observed in all age groups, thus emphasizing H_2_’s relevance as a rejuvenating and cytoprotective agent.

Despite these positive results, significant gaps remain. There is a lack of standardized clinical protocols for the application of molecular hydrogen topically, limited understanding of long-term effects, and a need for comprehensive biomarker-based studies to clarify the underlying mechanisms. It is recommended that future research include larger, controlled trials, a range of skin types, extended follow-up periods, and the integration of molecular diagnostics to validate therapeutic outcomes. In order to further define the role of H_2_ in evidence-based cosmetology and dermatology, it may be advisable to explore the dose–response relationship, formulation stability, and combination therapies with other antioxidants.

The present findings support the growing interest in molecular hydrogen as a non-invasive, well-tolerated approach to modulate oxidative skin damage. Further validation is required to determine the potential of H_2_-based therapies as a valuable addition to the repertoire of modern antioxidant and anti-aging interventions.

## Figures and Tables

**Table 1:** Low-mineral and hydrogen-rich water parameters comparison.

Parameter	Low-Mineral Water	Hydrogen-Rich Water
Temperature (°C)	21	27.2–28.3–29.2
pH	6.71	10.38
ORP (mV)	66	−212
H_2_ (ppb)	0	2071
H_2_ (ppm)	0	2.091

**Table 2:** Acidic and hydrogen-rich water parameters comparison.

Parameter	Acidic Water	Hydrogen-Rich Water
Temperature (°C)	30.8	26.9–27–28.4
pH	5.80–6.57	10.49
ORP (mV)	551/555	−204/−205
H_2_ (ppb)	0	2061/2063
H_2_ (ppm)	0	2.066

**Table 3:** Means and standard deviations for pores and porphyrins at three time points (baseline, immediately after treatment, and one week post-treatment) in three age groups (M-I, M-II, and M-III). Repeated measures ANOVA was conducted to evaluate within-group changes over time. F, p, and partial eta squared (ηp^2^) values indicate the statistical significance and magnitude of treatment effects over time. Abbreviations: M—mean; SD—standard deviation; F—F-ratio from one-way repeated measures ANOVA (time as within-subject factor); *p*—*p*-value for statistical significance (*p* < 0.05 considered significant); ηp^2^—partial eta squared, effect size estimate (0.01 = small, 0.06 = moderate, ≥0.14 = large effect). Note: For baseline values, F, *p*, and ηp^2^ were not calculated because these represent the reference point before treatment and are not subject to change over time.

Variable	M-I	SD	M-II	SD	M-III	SD	F	*p*	ηp^2^
Pores									
Baseline	66.86	8.45	40.50	24.56	44.50	-	-	-	-
After	74.14	10.95	43.50	26.66	47.00	17.42	5.01	0.026	0.455
Week 1	75.86	11.48	44.50	27.05	47.75	18.21	5.01	0.026	0.455
Porphyrins									
Baseline	83.00	5.45	80.25	3.30	85.00	-	-	-	-
After	91.00	6.19	88.75	3.59	94.25	2.50	1.28	0.313	0.176
Week 1	93.71	6.50	91.75	3.30	95.50	1.29	0.58	0.575	0.088

**Table 4:** Means and standard deviations for brown spots, red spots, and UV-induced hyperpigmentation at three time points (baseline, immediately after treatment, and one week post-treatment) in three groups. Repeated measures ANOVA was used to assess within-group changes over time. Values for F, *p*, and partial eta squared (ηp^2^) indicate the statistical significance and strength of the time effect. Abbreviations: M—mean; SD—standard deviation; F—F-ratio from one-way repeated measures ANOVA (time as within-subject factor); *p*—*p*-value for statistical significance (*p* < 0.05 considered significant); ηp^2^—partial eta squared, effect size estimate (0.01 = small, 0.06 = moderate, ≥0.14 = large effect). Note: For baseline values, F, *p*, and ηp^2^ were not calculated because these represent the reference point before treatment and are not subject to change over time.

Variable	M-I	SD	M-II	SD	M-III	SD	F	*p*	ηp^2^
Brown Spots									
Baseline	27.0	2.5	33.0	2.5	44.0	2.5	–	–	–
After	30.0	2.5	34.0	2.5	46.0	2.5	92.94	<0.001	0.7749
Week 1	31.0	2.5	35.0	2.5	47.0	2.5	172.64	<0.001	0.8648
Red Spots									
Baseline	28.0	2.5	32.0	2.5	43.0	2.5	–	–	–
After	29.0	2.5	33.0	2.5	44.0	2.5	181.00	<0.001	0.8702
Week 1	30.0	2.5	34.0	2.5	45.0	2.5	133.49	<0.001	0.8318
UV Hyperpigmentation									
Baseline	69.0	2.5	79.0	2.5	89.0	2.5	–	–	–
After	71.0	2.5	80.0	2.5	90.0	2.5	219.26	<0.001	0.8904
Week 1	72.0	2.5	81.0	2.5	91.0	2.5	158.57	<0.001	0.8545

**Table 5:** Summary of key studies on molecular hydrogen’s effects on skin and oxidative stress. This table indicates the evidence strength (preclinical, clinical, and review) and model/disease stage (in vitro, animal, human studies, and disease models).

Reference	Intervention	Observed Effects	Evidence Strength	Model/Disease Stage
Ohsawa et al. (2007) [5]	H_2_ administration (oral/inhalation)	Selective reduction in hydroxyl radicals and peroxynitrite, reduction in oxidative stress	Preclinical	In vitro, animal models
Artamonov et al. (2023) [6]	-	Systematized ideas about nature, characteristics, and mechanisms of molecular hydrogen action	Review	Various cell types, including stem cells
Bai et al. (2022) [13]	Hydrogen-rich water (HRW) for periodontal disease	HRW inhibits inflammation, oxidative stress, and bacterial activity; supports oral tissue health	Review	Summary of human and preclinical studies
Guan et al. (2020) [14]	H_2_ inhalation/patients with confirmed COVID-19	Alleviation of dyspnea and respiratory symptoms regardless of disease severity		Open-label multicenter trial
Chilicka et al. (2021) [15]	Topical H_2_/healthy women and women with acne	Reduced sebum, increased hydration; effective and safe for acne vulgaris	Clinical	Human studies
Guo and Zhang (2025) [35]	H_2_ in cosmetology	Overview of therapeutic mechanisms, clinical potential, and future perspectives	Review	Summary of human and preclinical studies
Tanaka and Miwa (2022) [16]	Repetitive H₂-rich bathing and skin poultice	Improvements in wrinkles, blotches, oiliness, and moisture	Clinical	Human studies
Tanaka et al. (2022) [24]	Hydrogen–water bathing	Enhanced antioxidant capacity, reduced inflammation, improved skin appearance	Clinical	Human study (dermatology)
Zhu et al. (2018) [25]	Hydrogen–water bathing	Improvement in psoriasis and parapsoriasis en plaques	Clinical	Human study (dermatology)
Hu et al. (2024) [26]	Hydrogen–water bathing	Improvement in atopic dermatitis	Clinical	Human study

**Table 6:** Means and standard deviations for overall skin condition, skin structure, and wrinkle severity at three time points (baseline, immediately after treatment, and one week post-treatment) in three age groups. Repeated measures ANOVA was used to assess within-group changes over time. Values for F, *p*, and partial eta squared (ηp^2^) indicate the statistical significance and strength of the time effect. Abbreviations: M—mean; SD—standard deviation; F—F-ratio from one-way repeated measures ANOVA (time as within-subject factor); *p*—*p*-value for statistical significance (*p* < 0.05 considered significant); ηp^2^—partial eta squared, effect size estimate (0.01 = small, 0.06 = moderate, ≥0.14 = large effect). Note: For baseline values, F, *p*, and ηp^2^ were not calculated because these represent the reference point before treatment and are not subject to change over time.

Variable	M-I	SD	M-II	SD	M-III	SD	F	*p*	ηp^2^
Skin Condition							F	*p*	ηp^2^
Baseline	69.0	1.00	67.2	1.71	67.8	2.06	–	–	–
After	74.0	2.65	72.0	2.65	73.0	3.61	5.50	0.0440	0.6279
Week 1	77.0	3.61	73.0	2.65	75.0	2.65	2.44	0.1681	0.2610
Skin Structure							F	*p*	ηp^2^
Baseline	61.1	4.41	55.0	8.76	61.5	3.70	–	–	–
After	67.0	2.5	59.0	2.5	67.0	2.5	4.22	0.0530	0.3130
Week 1	68.0	2.5	60.0	2.5	69.0	2.5	2.75	0.1170	0.1940
Wrinkles							F	*p*	ηp^2^
Baseline	61.9	10.73	51.8	9.91	58.2	10.53	–	–	–
After	67.0	2.5	55.0	2.5	63.0	2.5	3.89	0.0612	0.2882
Week 1	69.0	2.5	56.0	2.5	64.0	2.5	2.34	0.1380	0.1740

## Data Availability

The original contributions presented in this study are included in the article; further inquiries can be directed to the corresponding authors.

## Abbreviations

The following abbreviations are used in this manuscript:

ROS	reactive oxygen species
UV	ultraviolet
H_2_	molecular hydrogen
ORP	oxidation–reduction potential
TEWL	transepidermal water loss
MMPs	matrix metalloproteinases
Nrf2	nuclear factor erythroid 2-related factor 2
NF-κB	nuclear factor kappa-light-chain-enhancer of activated B cells
IL-1β,	interleukin-1 beta,
IL-6	interleukin-6
TNF-α	tumor necrosis factor alpha
AD	atopic dermatitis
ERW	electrolyzed reduced water
SD	standard deviation
η^2^ (ηp^2^)	partial eta squared (effect size)
RGB	red, green, blue (lighting system)
KLKs	kallikrein-related peptidases
